# Effects of boron and nitrogen doping on the electronic properties of graphene-based heterostructures with two-dimensional semiconducting materials

**DOI:** 10.1371/journal.pone.0348086

**Published:** 2026-05-08

**Authors:** Zhiang Liu, Ping Huang, Yi Luo, Pinbo Huang

**Affiliations:** 1 Chengdu Aeronautic Polytechnic University, Chengdu, China; 2 School of Mechanical Engineering, Jiangsu Ocean University, Lianyungang, China; National Research Centre, EGYPT

## Abstract

Based on first-principles density functional theory (DFT) calculations*,* heterostructures composed of graphene and two-dimensional semiconducting materials exhibit significant potential for applications in nanoelectronics devices. In this study, we propose an innovative approach to modulate the electronic properties of such heterostructures. We conduct a comprehensive investigation into the structural properties, band structures, and band alignments of ten widely studied two-dimensional semiconducting materials, unveiling opportunities for forming Schottky contacts with graphene. Furthermore, we demonstrate that the work function of graphene can be extensively tuned via boron or nitrogen doping. Our findings reveal that B- or N-doping in graphene substantially modifies the electronic properties of graphene/two-dimensional semiconducting materials heterostructures, enabling transitions between p-type and n-type Schottky contacts and even facilitating a shift from Schottky to Ohmic contacts in some cases. This tunability is critical for the precise design of graphene/two-dimensional semiconducting materials heterostructures with optimized Schottky barrier heights, paving the way for advanced nanoelectronics and catalyst applications.

## Introduction

Two-dimensional (2D) materials have attracted significant attention from researchers in both experimental and theoretical fields due to their remarkable properties [1–10]. Graphene, a 2D honeycomb lattice of carbon atoms, was the first such material discovered and remains the most extensively studied [11,12]. Its charge carriers exhibit zero effective mass and ballistic transport behavior at room temperature [1]. However, the wide applications of graphene have been limited by its intrinsic zero-bandgap semiconducting nature. Following the discovery of graphene, numerous 2D semiconducting materials (2DSMs), such as hexagonal boron nitride (h-BN), transition metal dichalcogenides (TMDs), phosphorene, and arsenene, have emerged and also attacted much attentions.

Recently, the construction of graphene-based heterostructures has been proposed as an innovative strategy to integrate the merits of graphene and 2DSMs. Various graphene-based heterostructures have been designed and explored, including graphene/MoS₂ [13], graphene/WSe₂ [14], graphene/black phosphorene [15], graphene/blue phosphorene [16], graphene/arsenene [17], graphene/SiC [18], graphene/GeC [19], and graphene/graphene-like-GaN (g-GaN) [20]. In these heterostructures, interactions between graphene and the semiconducting layers are dominated by van der Waals (vdW) forces. This interaction mechanism prevents Fermi-level pinning, enabling the design of 2D-material-based junctions for nanoeletronic devices with specific functionalities.

In addition to nanoelectronic devices, Schottky heterostructures can also serve as a critical platform for enhancing catalytic processes. Their significance lies in the ability to regulate charge transfer and dynamics at the metal-semiconductor interface, profoundly influencing the catalytic activity and selectivity of various reactions. Typical applications of Schottky heterostructures include photocatalysis (which improves charge separation efficiency and enhances redox reactions) and electrocatalysis (such as the hydrogen evolution reaction and CO_2_ reduction). The adjustment of the Schottky barrier allows for the control of the size and electron density of electron-rich and electron-depleted regions, thereby enabling precise tuning of the overall redox capacity of the catalyst and reaction rates. Therefore, achieving control over the Schottky barrier in Schottky heterostructures is of great significance [21].

Despite their excellent properties and potential applications, some fundamental questions remain unanswered for graphene/2DSM heterostructures. For instance, due to low density of states at the Fermi level, the work function of graphene is highly tunable. While various methods have been applied to tune its work function, boron and nitrogen doping are among the most widely adopted techniques [22]. These dopants significantly tune the electronic properties of graphene-based heterostructures. Thus, a systematic understanding of the effects of boron and nitrogen doping on the electronic properties of such heterostructures remains largely unknown and incomplete. Furthermore, although previous studies have mainly focused on a few representative systems, they often limited to qualitative analyses of charge transfer or band alignment under specific conditions. A systematic comparison across multiple two-dimensional semiconductors with diverse orbital characteristics (e.g., transition metal dichalcogenides, pnictogens, and wide-bandgap systems) is still lacking.

In this work, we provide a unified doping-based tuning framework by systematically comparing ten different 2D semiconductors, focusing on their band edge positions and their potential to form heterostructures with graphene. We demonstrate how boron and nitrogen doping in graphene modulates Schottky barrier heights and induces transitions between Schottky and Ohmic contacts, offering generalized insight into contact engineering in 2D heterostructures. Our calculations reveal that such doping significantly influences the electronic properties of the heterostructure, providing valuable insights for the design of advanced nanoelectronic devices with certain functions.

## Results and discussion

**Fig 1:**
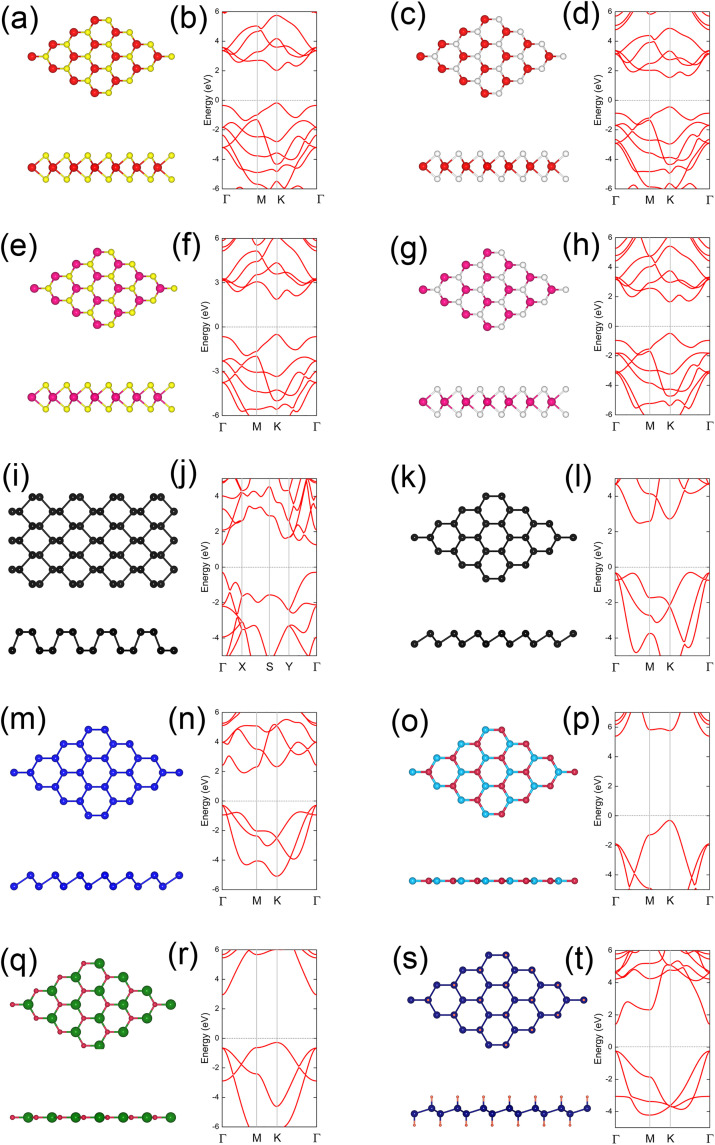
Crystal structures and electronic band structures (the Fermi level is set to zero) of the ten two-dimensional semiconductors studied: (a,b) MoS_₂_, (c,d) MoSe_₂_, (e,f) WS_₂_, (g,h) WSe_₂_, (i,j) black phosphorene, (k,l) blue phosphorene, (m,n) arsenene, (o,p) h-BN, (q,r) g-GaN, and (s,t) germanane.

We began by examining the structural and electronic properties, as well as the band edge positions, of several widely studied 2DSMs, e.g., MoS₂, MoSe₂, WS₂, WSe₂, black phosphorene, blue phosphorene, arsenene, h-BN, g-GaN, and germanane. Among these materials, MoS₂, MoSe₂, WS₂, WSe₂, blue phosphorene, arsenene, h-BN, g-GaN, and germanane share a hexagonal honeycomb structure similar to that of graphene, as illustrated in Fig 1a, 1(c), 1(e), 1(g), 1(k), 1(m), 1(o), 1(q), 1(s), and 1(u), respectively. In contrast, black phosphorene adopts a puckered honeycomb structure, as shown in Fig 1i. Based on our calculations, the lattice parameters of MoS₂, MoSe₂, WS₂, WSe₂, blue phosphorene, arsenene, h-BN, g-GaN, and germanane are 3.16, 3.29, 3.17, 3.29, 3.27, 3.60, 2.51, 3.25, and 4.08 Å, respectively (see S1 Table). Meanwhile, the lattice constants for black phosphorene are *a* = 4.60 Å and *b* = 3.30 Å. These results align well with previously reported values, confirming the reliability of our calculations [23].

Atom colors: red (Mo), yellow (S), white (Se), magenta (W), black (P), blue (As), deep sky-blue (B), crimson (N), sea-green (Ga), navy (Ge), and tomato-red (H).

The band structures categorize these 2DSMs into two groups. The first group includes MoS₂, MoSe₂, WS₂, WSe₂, black phosphorene, and germanane, which are all direct-bandgap semiconductors, as illustrated in Fig 1b, 1(d), 1(f), 1(h), 1(j), and 1(t). Their bandgaps are 2.20, 1.99, 2.37, 2.12, 1.56, and 1.66 eV, respectively (see S1 Table). The second group contains blue phosphorene, arsenene, h-BN, and g-GaN, which are indirect-bandgap semiconductors, as shown in Fig 1l, 1(n), 1(p), and 1(t). The bandgaps of these materials are 2.77, 2.21, 5.71, and 3.23 eV, respectively. These bandgap values are consistent with previous reports [23], further validating the accuracy of our calculations.

**Fig 2:**
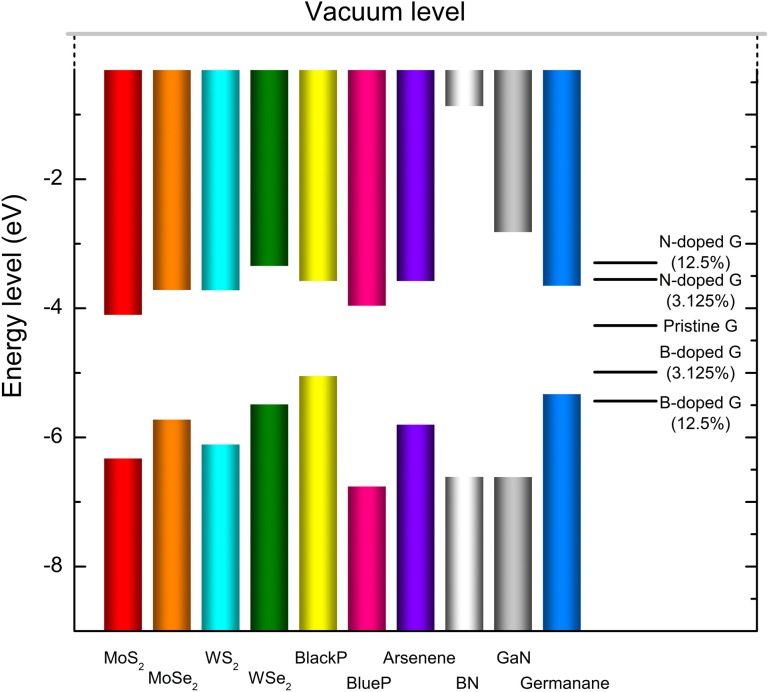
Band alignments of MoS₂, MoSe₂, WS₂, WSe₂, black phosphorene, blue phosphorene, arsenene, h-BN, g-GaN, and germanane with graphene, B-doped graphene, and N-doped graphene.

The band alignments of MoS₂, MoSe₂, WS₂, WSe₂, black phosphorene, blue phosphorene, arsenene, h-BN, g-GaN, and germanane are presented in Fig 2. The conduction band minimum (CBM) values for these materials are −4.114, −3.730, −3.734, −3.356, −3.483, −3.972, −3.589, −0.897, −2.833, and −3.663 eV, respectively. Meanwhile, their valence band maximum (VBM) values are −6.318, −5.716, −6.101, −5.478, −5.039, −6.746, −5.794, −6.603, −6.065, and −5.321 eV, respectively. Additionally, the work function of graphene, one of the most studied Dirac materials, is included for reference.

In graphene/2DSM heterostructures, the interaction between the graphene and the 2DSM layers is predominantly governed by vdW forces, which preserve the electronic properties of the individual layers. Depending on the work function of graphene and the band edge positions of the 2DSM, the graphene layer can form either a Schottky or an Ohmic contact with the 2DSM layer. For Schottky contacts, the Schottky barrier height (SBH) can be determined using the Schottky–Mott rule [24,25]. Specifically, the n-type SBH is defined as the energy difference between the CBM of the 2DSM and the Dirac point, while the p-type SBH is defined as the energy difference between the VBM of the 2DSM and the Dirac point of graphene. If the n-type SBH is smaller than the p-type SBH, the heterostructure forms a n-type Schottky contact, and vice versa. Based on these definitions, our calculations suggest that graphene/MoS₂, graphene/MoSe₂, graphene/WS₂, graphene/WSe₂, graphene/blue phosphorene, graphene/arsenene, graphene/h-BN, graphene/g-GaN, and graphene/germanane vdW heterostructures are likely to form n-type Schottky contacts. In contrast, graphene/black phosphorene and graphene/h-BN vdW heterostructures are more likely to form p-type Schottky contacts.

**Fig 3:**
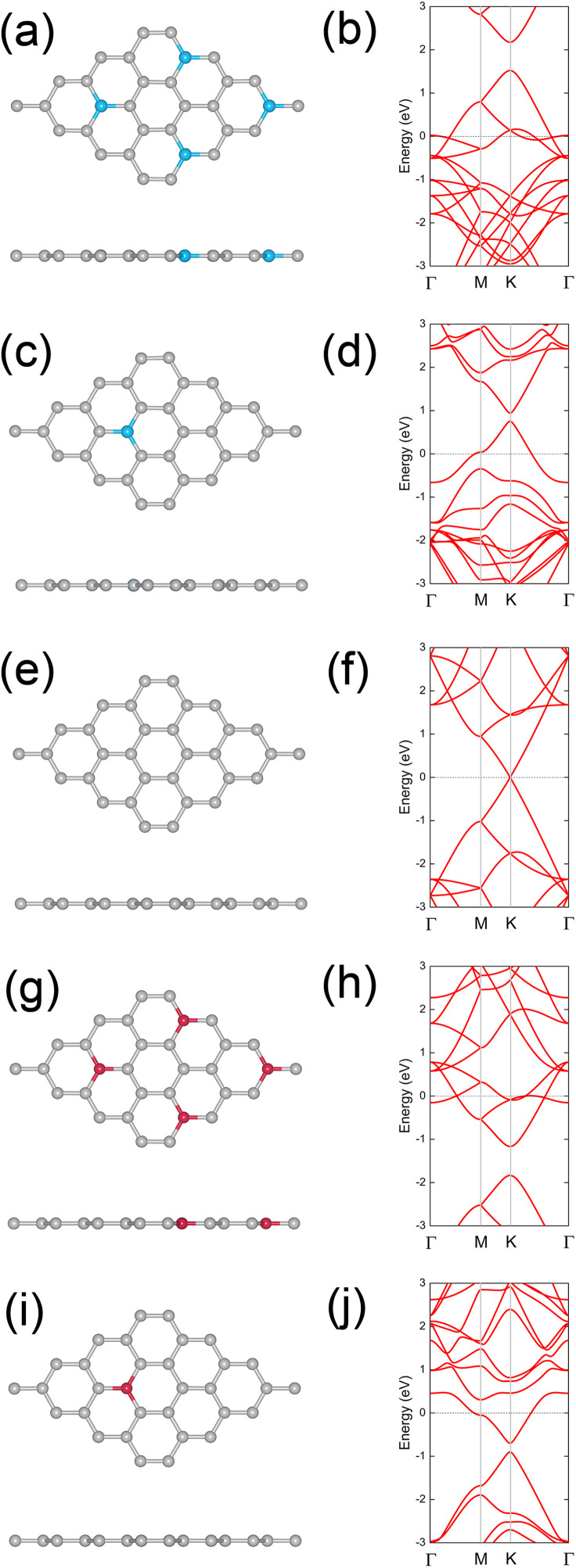
Crystal structures of (a) B-doped graphene (dopant concentration: 12.5%), (c) B-doped graphene (dopant concentration: 3.125%), (e) pristine graphene, (g) N-doped graphene (dopant concentration: 12.5%), and (i) N-doped graphene (dopant concentration: 3.125%). The grey, blue, and red spheres represent C, B, and N atoms, respectively. Corresponding band structures with the Fermi level set to zero energy are shown in (b), (d), (f), (h), and (j), respectively.

Previous research has employed various strategies to tune the SBH of Schottky contacts, such as modifying interlayer coupling, applying external electric fields, and introducing adsorbates. In this study, we explored the effects of boron (B) and nitrogen (N) doping on graphene as a method to tune the SBH in graphene/2DSM heterostructures. We first analyzed the electronic properties of B- and N-doped graphene. The crystal structures of B-doped graphene with doping concentrations of 3.125% and 12.5% are shown in Fig 3(a) and Fig 3(c), respectively, with their corresponding band structures depicted in Fig 3(b) and Fig 3(d). For comparison, the crystal structure and band structure of pristine graphene are provided in Fig 3(e) and Fig 3(f). The results indicate that B-doping significantly lowers the Fermi level of graphene, consistent with its p-type doping behavior. Similarly, the crystal structures of N-doped graphene with doping concentrations of 3.125% and 12.5% are shown in Fig 3(g) and Fig 3(i), while the corresponding band structures are presented in Fig 3(h) and Fig 3(j). N-doping raises the Fermi level of graphene, confirming its n-type doping nature. The work functions of B- and N-doped graphene systems are summarized in Fig 2. The work functions for B-doped graphene at 3.125% and 12.5% concentrations are −4.996 and −5.439 eV, respectively. In contrast, the work functions for N-doped graphene at 3.125% and 12.5% concentrations are −3.559 and −3.301 eV, respectively. These results demonstrate that the Fermi level of graphene can be effectively tuned within a wide range through B- or N-doping, providing a versatile approach for tuning the electronic properties of graphene/2DSM heterostructures.

The distinct effects of B and N dopants originate from their different electronic configurations relative to carbon. Boron having one fewer valence electron than carbon, acts as an electron acceptor and introduces hole-like states in the graphene π-network, resulting in a downward shift of the Fermi level and p-type behavior. In contrast, nitrogen possessing one additional valence electron, serves as an electron donor, injecting electrons into the π-network and raising the Fermi level, which corresponds to n-type doping. These opposite doping effects modulate the interface dipole and thus the potential lineup at the heterointerface, effectively tuning the Schottky barrier height.

The tunability of graphene’s Fermi level over a wide range significantly affects the SBH in graphene/2DSM heterostructures, as demonstrated in Fig 2. For example, graphene can form a p-type Schottky contact with black phosphorene, while doping graphene with boron or nitrogen can transform this interface into an Ohmic contact. Besides, we find an n-type Schottky contacts formed at the graphene/blue phosphorene interface. We further find B-doped graphene (3.125%) facilitates a transition from n-type to p-type Schottky contact, while N-doped graphene (3.125%) can lead to a transition from n-type Schottky to Ohmic contact in the graphene/blue phosphorene heterostructure.

**Fig 4:**
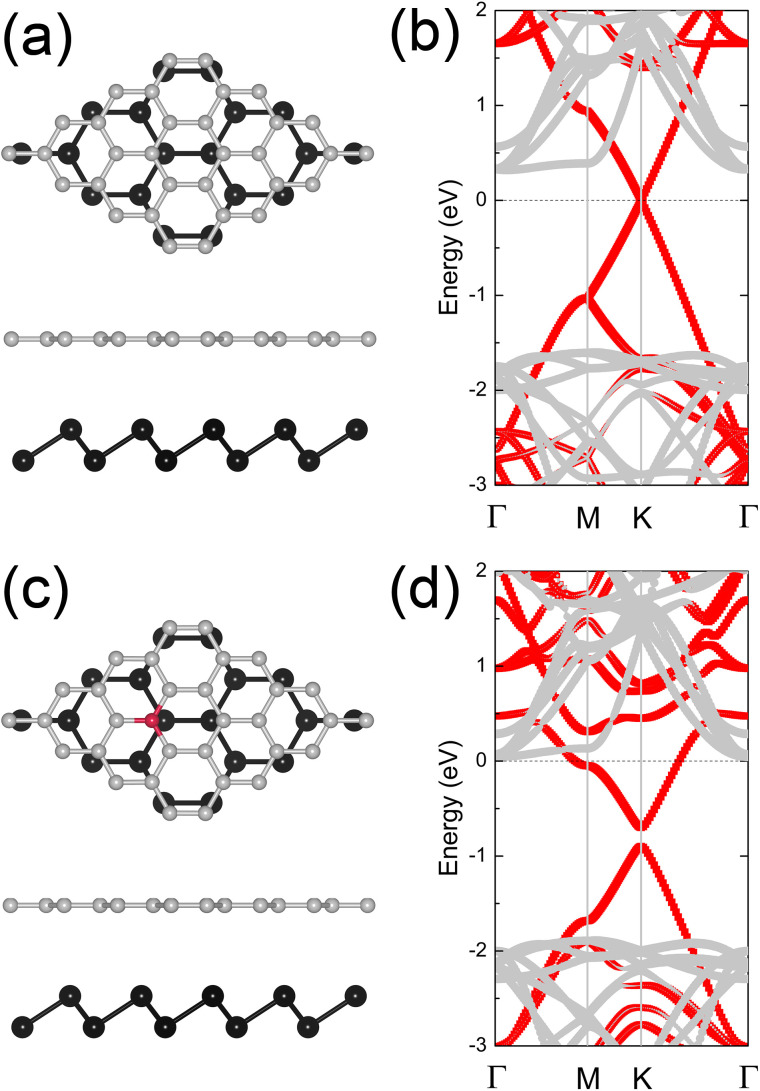
Crystal structures of (a) graphene/blue phosphorene and (c) N-doped graphene/blue phosphorene vdW heterostructures. Grey, red, and black spheres represent C, N, and P atoms, respectively. Projected band structures for these heterostructures are shown in (b) and (d), with grey and red symbols representing the contributions from graphene (including doped graphene) and blue phosphorene, respectively. The Fermi level is set to zero.

To confirm this, we fully relaxed the crystal structures and calculate the projected band structures of graphene/blue phosphorene and N-doped graphene/blue phosphorene heterostructures. For constructing the heterostructures, the graphene and blue phosphorene were matched using small commensurate supercells (4 × 4 graphene combined with 3 × 3 blue phosphorene), leading the lattice mismatch only 0.5%. In Fig 4(a), the crystal structure of graphene/blue phosphorene is shown, with its projected band structure presented in Fig 4(b). The Dirac cone of graphene is near the conduction band minimum (CBM) of blue phosphorene, resulting in an n-type SBH of 0.33 eV, which is smaller than the p-type SBH of 1.60 eV, confirming an n-type Schottky contact. This result aligns with predictions from Fig 2. For the N-doped graphene/blue phosphorene heterostructure, as shown in Fig 4(c) and 4(d), N-doping shifts the Fermi level near the CBM of blue phosphorene, resulting in a transition from n-type Schottky contact to an n-type Ohmic contact, even at a dopant concentration of only 3.125%. These findings are also consistent with predictions from Fig 2.

## Conclusions

In summary, we systematically investigated the structural and electronic properties, as well as band alignment of graphene/2DSM heterostructures, exploring their potential to form Schottky contacts and the tunability enabled by boron and nitrogen doping. Our findings reveal that graphene can form either n-type or p-type Schottky contacts with various 2DSM. By employing B and N doping, the Fermi level of graphene can be tuned to enable transitions between n-type and p-type Schottky contacts and even achieve a transition from Schottky to Ohmic contacts. This provides a powerful approach for modulating Schottky barrier heights, allowing precise control over the electronic properties and carrier dynamics of heterostructures. This tunability is not only critical for applications of graphene/2DSM heterostructures in nanoelectronics but also plays a pivotal role in controlling the catalytic activity and reaction rates in catalytic systems. Our study highlights the potential of doping strategies in designing advanced functional heterostructures, paving the way for innovations in nanoelectronics, optoelectronics, and catalysis. Beyond the comparative DFT framework, there is growing experimental evidence that doping graphene electrodes can drastically reduce contact resistance and even yield near-Ohmic behavior in 2D devices. For example, Seo *et al.* employed nitrogen-doped graphene to contact monolayer MoS₂ and achieved barrier-free Ohmic contact at modest gate bias with >200% on-current enhancement and four-times mobility improvement, directly linking graphene doping to Schottky barrier suppression [26]. This agreement indicates that the present theoretical framework captures the essential physics governing doping-induced contact tunability in 2D heterostructures. Our investigation not only contribute to understanding the tunability of graphene based Schottky contact by doping but also provide guidelines for future experimental research on nanoelectronics and catalysts.

## Methods

First-principles calculations were performed using the Vienna *Ab Initio* Simulation Package [27], based on density functional theory with a plane-wave basis set and the projector-augmented wave method [28]. The generalized gradient approximation of Perdew, Burke, and Ernzerhof was employed to describe the exchange-correlation functional, with the Heyd–Scuseria–Ernzerhof hybrid functional [29] for obtaining accurate electronic properties and to correct for the well-known band-gap underestimation of standard GGA calculations. vdW interactions were treated using the DFT-D3 method with zero-damping [30]. Plane-wave expansion was set with an energy cutoff of 550 eV, and the *k*-point density denser than 2π × 0.03 Å^−1^ with Monkhorst–Pack scheme [31] was applied for Brillouin zone sampling. A vacuum thickness of 20 Å was used to eliminate interactions between periodic images. Full structural relaxations were performed until atomic forces were below 0.01 eV/Å. Spin–orbit coupling (SOC) was not included in all calculations.

## Supporting information

S1 Table Structural and electronic properties of the 2D semiconductors investigated. All values were obtained from HSE06 calculations. Zero energy is referred to the vacuum level.
(DOCX)
