# Supplementary material for: Effects of boron and nitrogen doping on the electronic properties of graphene-based heterostructures with two-dimensional semiconducting materials
Source: PLoS One. 2026 May 8;21(5):e0348086. doi: 10.1371/journal.pone.0348086 (PMC13155675; doi:10.1371/journal.pone.0348086)
Supplement: S1 Table — All values were obtained from HSE06 calculations. Zero energy is referred to the vacuum level. (DOCX) [file pone.0348086.s001.docx]

**Supporting Information for**

**Effects of Boron and Nitrogen Doping on the Electronic Properties of Graphene-Based Heterostructures with Two-Dimensional Semiconducting Materials**

Zhiang Liu,^1^ Ping Huang,^1^ Yi Luo,^2^ and Pinbo Huang^1,^*

*1. Chengdu Aeronautic Polytechnic University, Chengdu 610100, China*

*2. School of Mechanical Engineering, Jiangsu Ocean University, Lianyungang 222005, China*

**Correspondence:* [*pinbohuang@cap.edu.cn*](mailto:pinbohuang@cap.edu.cn) *(Pinbo Huang)*

Supporting Information Table 1. Structural and electronic properties of the 2D semiconductors investigated. All values were obtained from HSE06 calculations. Zero energy is referred to the vacuum level.

| Material | Lattice constant (Å) | Bandgap (eV) | CBM (eV) | VBM (eV) |
| --- | --- | --- | --- | --- |
| MoS₂ | 3.16 | 2.20 | −4.114 | −6.318 |
| MoSe₂ | 3.29 | 1.99 | −3.730 | −5.716 |
| WS₂ | 3.17 | 2.37 | −3.734 | −6.101 |
| WSe₂ | 3.29 | 2.12 | −3.356 | −5.478 |
| Black phosphorene | 4.60/3.30 | 1.56 | −3.483 | −5.039 |
| Blue phosphorene | 3.27 | 2.77 | −3.972 | −6.746 |
| Arsenene | 3.60 | 2.21 | −3.589 | −5.794 |
| h-BN | 2.51 | 5.71 | −0.897 | −6.603 |
| g-GaN | 3.25 | 3.23 | −2.833 | −6.065 |
| Germanane | 4.08 | 1.66 | −3.663 | −5.321 |
